# Clinical outcome, quality of life, and mental health in long-gap esophageal atresia: comparison of gastric sleeve pull-up and delayed primary anastomosis

**DOI:** 10.1007/s00383-023-05448-4

**Published:** 2023-04-04

**Authors:** Michael Boettcher, Marie Hauck, Mareike Fuerboeter, Julia Elrod, Deirdre Vincent, Johannes Boettcher, Konrad Reinshagen

**Affiliations:** 1 Department of Pediatric Surgery, University Medical Center Hamburg-Eppendorf, Hamburg, Germany; 2 Department of Pediatric Surgery, University Medical Center Mannheim, University of Heidelberg, Theodor-Kutzer-Ufer 1-3, 68167 Mannheim, Germany; 3 Department of Child and Adolescent Psychiatry, Psychosomatics and Psychotherapy, University Medical Center Hamburg-Eppendorf, Hamburg, Germany

**Keywords:** Esophageal atresia, Gastric pull-up, Long gap, Delayed primary anastomosis

## Abstract

**Introduction:**

Pediatric surgeons have yet to reach a consensus whether a gastric sleeve pull-up or delayed primary anastomosis for the treatment of esophageal atresia (EA), especially of the long-gap type (LGEA) should be performed. Thus, the aim of this study was to evaluate clinical outcome, quality of life (QoL), and mental health of patients with EA and their parents.

**Methods:**

Clinical outcomes of all children treated with EA from 2007 to 2021 were collected and parents of affected children were asked to participate in questionnaires regarding their Quality of Life (QoL) and their child’s Health-Related Quality of Life (HRQoL), as well as mental health.

**Results:**

A total of 98 EA patients were included in the study. For analysis, the cohort was divided into two groups: (1) primary versus (2) secondary anastomosis, while the secondary anastomosis group was subdivided into (a) delayed primary anastomosis and (b) gastric sleeve pull-up and compared with each other. When comparing the secondary anastomosis group, significant differences were found between the delayed primary anastomosis and gastric sleeve pull-up group; the duration of anesthesia during anastomosis surgery (478.54 vs 328.82 min, *p* < 0.001), endoscopic dilatation rate (100% vs 69%, *p* = 0.03), cumulative time spent in intensive care (42.31 vs 94.75 days, *p* = 0.03) and the mortality rate (0% vs 31%, *p* = 0.03). HRQoL and mental health did not differ between any of the groups.

**Conclusion:**

Delayed primary anastomosis or gastric sleeve pull-up appear to be similar in patients with long-gap esophageal atresia in many key aspects like leakage rate, strictures, re-fistula, tracheomalacia, recurrent infections, thrive or reflux. Moreover, HrQoL was comparable in patients with (a) gastric sleeve pull-up and (b) delayed primary anastomosis. Future studies should focus on the long-term results of either preservation or replacement of the esophagus in children.

**Supplementary Information:**

The online version contains supplementary material available at 10.1007/s00383-023-05448-4.

## Introduction

Esophageal Atresia (EA) is a rare congenital malformation occurring in every 3–4 per 10.000 births [1]. In Germany less than 170 children undergo EA repair each year [2]. In particular, long-gap EA (LGEA), a form of EA with a large distance between the atretic ends, remains a rare and challenging condition for pediatric surgeons to treat.

While survival has dramatically improved over the last several decades, morbidity remains high [3–5]. Although no real consensus on the definition of LGEA exists, it is agreed upon that most LGEA cases cannot be corrected using a primary anastomosis of the esophageal ends [4]. In these cases, a secondary anastomosis surgical approach is required, which either involves (a) a delayed repair (delayed primary anastomosis) or (b) replacement (i.e., gastric pull-up or intestinal interposition) [6].

Proponents of the first method described above, namely repair, postulate that the native esophagus is the optimal conduit for EA repair and consequently esophageal preservation should be the primary goal of surgical management [4, 6]. As such, various techniques have been described involving primary anastomosis: elongation of the esophagus via (1) extrathoracically (Kimura technique), (2) external (Foker technique), or (3) internal traction sutures (Patkowski technique) [7, 8]. Even though replacement and preservation co-exist, experts have not been able to determine whether a delayed primary anastomosis, following traction of the esophagus, is superior to a replacement involving gastric, small intestinal or colonic interposition [9, 10]. As such, although there is a growing body of research on the long-term outcomes of individuals affected by EA, data comparing delayed primary anastomosis and gastric sleeve pull-up have been missing.

To assess which surgical method might be superior and to find consensus amongst experts, post-operative complications such as stricture rate, weight gain and the presence of reflux or dysphagia should be taken into account in the assessment of patient outcome, as recent studies have found conflicting results. Hannon et al. showed that children with gastric sleeve pull-up had significantly lower weight, higher need for supplementary feeding (19 vs. 0%), and dumping symptoms (25 vs. 0%) in adulthood [11], while other authors stated no significant differences in complications, length of hospital stay, or weight gain when comparing both techniques in long-gap EA patients [10].

However, as shown in current studies involving other congenital malformations, it is not sufficient to only assess clinical outcomes, such as the number and severity of complications, but one should also consider the subjective health-related quality of life (HRQoL) of the child and Quality of Life (QoL) of its parents, to determine whether one particular procedure is ‘superior’ [12] Mental health, can be defined as the “flexibility and ability to cope with adverse life events and function in social roles” [13], whereas QoL can be described as “the individuals’ perception of their position in life in the context of the culture and value systems in which they live, in relation to their goals, expectations, standards and concerns” [14]. Recent reports suggest that mental health and HRQoL is only partially affected in children and adolescent with EA patients. More precise, EA patient seem to have emotional and behavioral problems when compared to the normative population [15–18]. No differences in the child’s HRQoL, between short and long gaps, have been reported [11, 19, 20]. However, families of children with EA seem to be burdened and recent studies reported a significantly reduced (Hr)QoL for these families [21, 22].

Knowledge of outcomes in the two popular secondary anastomoses techniques, namely (a) delayed primary repair and (b) gastric sleeve pull-up, focusing on patient’s clinical outcome and psychosocial condition (HrQoL and mental health) is needed. Thus, the aim of this study was to compare clinical outcomes, HRQoL, and mental health of children with esophageal atresia who underwent gastric sleeve pull-up to those who underwent delayed primary anastomosis surgery. In addition, following research questions were addressed: Do significant differences exists (1) in the distribution of clinical variables, HRQoL, and mental health between the primary anastomosis cohort and secondary anastomosis cohort, (2) in the distribution of HRQoL and mental health of the parents of affected children, and (3) in the distribution of HRQoL and mental health between affected patients and norm data?

## Methods

### Study design

The study included all children who underwent surgery for EA repair at the University Medical Center Hamburg-Eppendorf between April 2007 and April 2021. The follow-up was based on the protocol of the Esophageal Atresia and Tracheo-Esophageal Fistula Support Federation (KEKS). This includes follow-up visits at the age of 6 months, 1 year, 2 years, 4 years, 6 years, 10 years, 14 years and at age 18. Weight, food intake and reflux symptoms are checked at every visit. Clinical features are reevaluated until adulthood. Patients with missing data, such as long-term follow ups or those who refused to participate in the study, were excluded. The study received ethical approval from the Medical Chamber Hamburg (PV7161) and was registered at ClinicalTrials.gov (NCT04382820). Analysis and reporting were guided by Strengthening the Reporting of Observational Studies in Epidemiology (STROBE) recommendations [23]. The patients were grouped into two main cohorts: (1) primary and (2) secondary anastomosis, while the secondary anastomosis group comprised the subgroups (a) gastric sleeve pull-up and (b) delayed primary anastomosis, which are the two main operating techniques performed for LGEA at our medical center.

### Clinical variables

Patient data were collected using medical records and included clinical variables such as details regarding perinatal data, birth weight/length, and co-morbidities (i.e., VACTERL). The type and length of esophageal gap were identified by review of initial postnatal chest X-rays: The type of EA was defined according to VOGT criteria. Additionally, LGEA was defined as a gap between the proximal and distal esophageal ends measuring ≥ 3 vertebrae.

Moreover, age and weight at the time of EA corrective surgery, as well as the duration and type of surgery were analyzed. Post-operative data obtained for the study included duration of mechanical ventilation, chest tube, and length of stay in the intensive care unit (ICU). Common complications, both long and short term (dysphagia, reflux, tracheomalacia, strictures, leakage, PPI requirements), were noted. Further, details regarding the number and duration of hospitalizations pertaining to dilatation procedures were recorded.

With respect to weight gain data, feeding regimes before and after anastomosis were analyzed and the functional oral intake scale (FOIS) was used to evaluate oral intake. FOIS consists of a numeric scale quantifying oral intake, ranging from 1 (nothing by mouth) to 7 (full oral diet, no restrictions) [24]. Weight and height measurements were collected and converted into the weight-for-length *z*-score using The Netherlands Organization for Applied Scientific Research (TNO) growth standards [25]. To assess the presence of reflux symptoms, guardians were sent the ‘GERD-Q’; a questionnaire containing 7 items related to the frequency and severity of symptoms related to reflux disease [26, 27].

### Psychosocial outcomes

A study-specific questionnaire was sent to the families of affected children to assess the psychosocial outcomes like their (Hr)QoL and mental health status. Sociodemographic variables included sex, age, level of care required for the affected child, marital status, number of children, educational qualifications, employment status, and current physical/mental health status.

#### Parental quality of life (QoL)

Parental QoL was measured using the Ulm Quality of Life Inventory for Parents (ULQIE), which is designed for parents of chronically ill children [28]. The ULQIE consists of 29 items, which are rated on a five-point rating scale. Five respective subscales measure (1) physical and daily functioning, (2) satisfaction with the family, (3) emotional distress, (4) self-development, and (5) well-being. Solely, the total scale by averaging all score was used in this study, illustrating overall QoL. Lower scores indicate decreased QoL. The ULQIE has been shown to provide reliable psychometric properties and normative data for parents of chronically ill children suffering from various diseases [28].

#### Parental mental health

The parent’s mental health was measured with the self-report questionnaire Brief Symptom Inventory (BSI) [29]. The BSI consists of 53 items, measuring nine subscales including (1) somatization, (2) compulsivity, (3) interpersonal sensitivity, (4) depression, (5) anxiety, (6) hostility, (7) phobic fear, (8) paranoid thinking, and (9) psychoticism, and three global indices, including the Positive Symptom Distress Index, Positive Symptom Total, and Global Severity Index (GSI). In this study, solely the GSI was used to provide a composite score of overall distress by using the mean of all items. Lower scores indicate decreased mental health. The German version of the BSI has been found to assess psychometric properties of individuals in a reliable and valid fashion [29].

#### Children’s health-related quality of life

The children’s HRQoL using the parent-report version of the Pediatric Quality of Life Inventory—Short Form 15 (PedsQL TM 4.0 SF-15) [30]. The instrument consists of 15 items, measuring four subscales including (1) physical functioning, (2) emotional functioning, (3) social functioning, and (4) school functioning. Additionally, a total score can be calculated. Raw scores were converted into a standardized 0–100 scale according to the manual. In this study, solely the total score was used, with higher scores representing greater overall HRQoL. The German version of the PedsQL has shown adequate psychometric properties [31].

#### Children’s mental health

Children’s mental health was assessed using the Strength and Difficulties Questionnaire (SDQ) [32]. The SDQ consists of 25 items, which are rated on a three-point rating scale. The instrument comprises five subscales of 5 items each including (1) emotional symptoms, (2) conduct problems, (3) hyperactivity, (4) peer problems, (5) prosocial behavior, and a total difficulties score. Solely the total difficulties score was used, by summing scores from all scale, except the prosocial scale. The SDQ has been shown to assess emotional and behavioral status, as well as prosocial behavior [32].

### Statistics

For descriptive data, frequencies, means, standard deviations, and bivariate tests (Chi-square tests) were used. Differences between groups were calculated using *t*-tests or Wilcoxon Rank test. Pearson correlations was applied to investigate the bivariate associations between psychosocial outcomes. To account for any known biases, propensity score matching was performed using an optimal matching algorithm with a caliper of 0.2 for gender, age, weight at operation, diagnosis, and revisionary surgery. Multiple linear regression models were used to define predictors of psychosocial outcomes. To indicate the size of the effect, Cohen’s *d* and Cramer’s *V* were calculated. Statistical significance was set at *p* $\le$ 0.05 (two-tailed). Statistical analyses were conducted using SPSS Statistics 28 and GraphPad Prism 9.

## Results

**Fig. 1:**
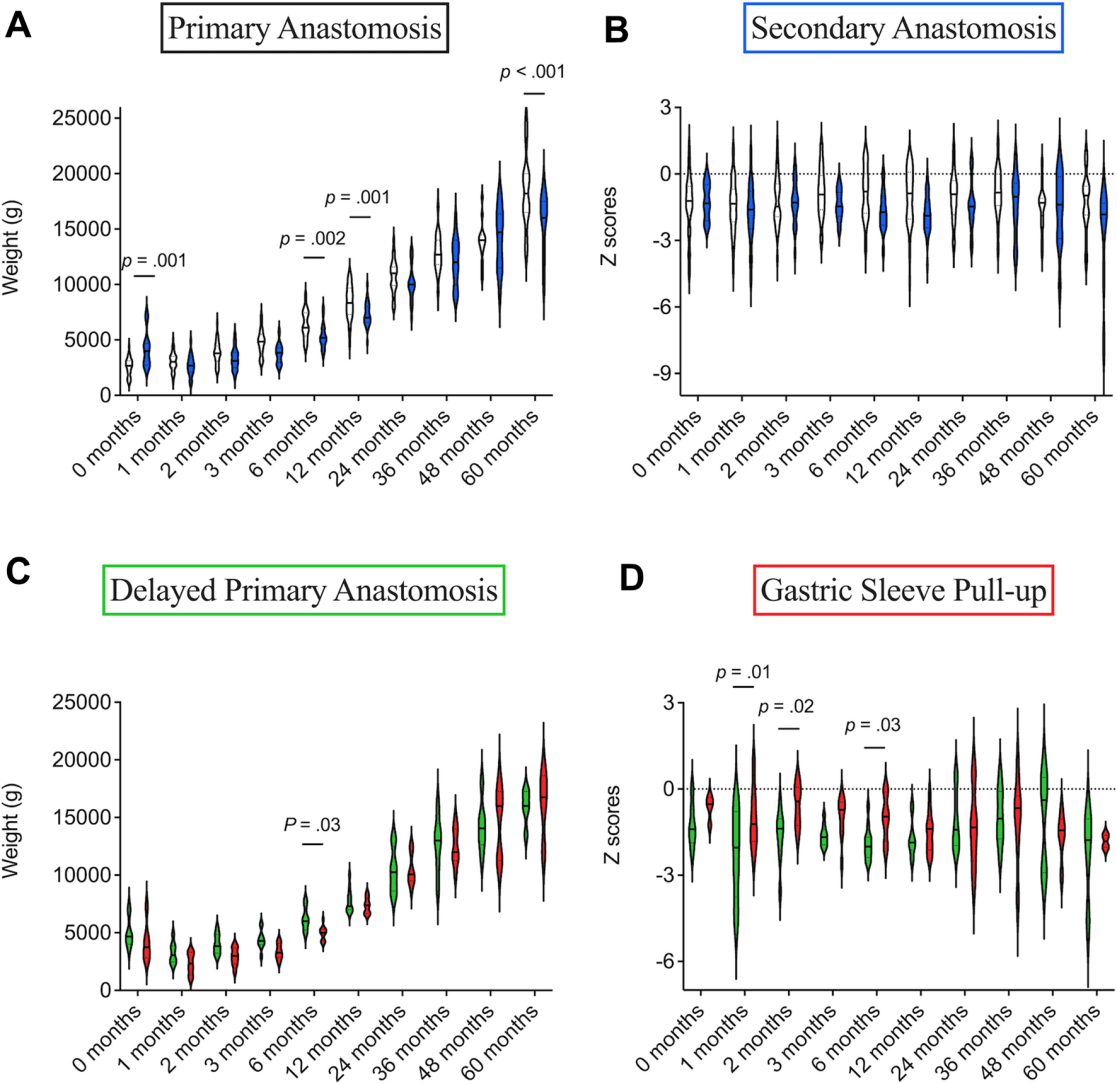
Distribution of weight-for-age and z-scores for primary and secondary anastomosis groups (**A**, **B**). Distribution of weight-for-age and z-scores for delayed primary anastomosis and gastric sleeve pull-up (**C**, **D**). Comparison between groups was conducted using ANOVA: Dunnets correction or *t*-test

In total, 104 children with EA were identified, of which 6 were excluded based on the previously stated exclusion criteria (Fig. 1). Overall, 61 patients underwent primary anastomosis for corrective surgery of EA, while 37 patients underwent a secondary anastomosis surgery. Out of the secondary anastomosis procedure, 24 patients were treated using the delayed primary anastomosis, which involved placement of internal traction sutures at the time of fistula ligation and a gastrostomy for feeding. The remaining 13 patients in the secondary anastomosis group received a gastric sleeve pull-up operation. Supplementary Fig. 1 shows the CONSORT flow diagram illustrating the total patient cohort and subgroups for analysis of clinical outcomes.

### Clinical features of primary vs. secondary anastomosis

Clinical outcomes of patients who underwent a primary anastomosis surgery were compared to those who underwent a secondary anastomosis surgery for EA. As suspected and shown in Table 1, children with primary anastomosis were significantly older, heavier, and had significantly shorter gap length than patients who underwent secondary repair. As shown in Table 2, primary anastomosis patients experienced significantly shorter operating and anesthesia times, during both the anastomosis repair surgery as well as all subsequent surgeries associated with the EA. However, the length of post-operative stay in the ICU and mechanical ventilation did not differ significantly between the primary and secondary anastomosis groups. Further, children with primary anastomoses had significantly fewer complications like tracheomalacia and reflux symptoms and were quicker to reach full oral feedings. Compared to patients who underwent secondary anastomosis, a primary anastomosis seems to lead to a fewer number of required endoscopic dilation procedures at the esophageal stricture site. Consequently, this results in a shorter cumulative duration of general anesthesia, operating time, and stay in the ICU.

**Table 1:** Distribution of disease characteristics of patients who underwent repair via secondary anastomosis: delayed primary anastomosis or gastric sleeve pull-up

	Delayed primary anastomosis *(n = *13*)*	Gastric sleeve pull-up *(n = *13*)*	*p*
Disease characteristics	Mean (SD)	Mean (SD)	
Demographics			
Gender (female)	46%	46%	n.s.
Gestation week	31.54 (4.18)	36.15 (2.57)	.002
Birth weight (grams)	1535.08 (805.26)	2507.31 (702.98)	.003
Birth weight (z-score)	− 1.00 (0.99)	− 0.89 (1.17)	n.s.
Body length (centimeter)	40.94 (5.39)	46.60 (3.17)	.01
Body length (z-score)	− 0.62 (0.97)	− 0.99 (0.85)	n.s.
Gemini	31%	15%	n.s.
Preterm (<36 gestational week)	92%	54%	.02
Cesarean-section	100%	69%	n.s.
Family history of esophageal atresia	0%	0%	n.s.
Presence of associated malformations			
Cardiac malformation	62%	23%	.05
Anorectal malformation	38%	0%	.01
Urogenital malformation	31%	15%	n.s.
Gastrointestinal malformation	23%	8%	n.s.
Musculoskeletal malformation	23%	15%	n.s.
Esophageal atresia			
Distance measured between proximal and distal ends (vertebrae)	3.73 (1.22)	4.73 (1.67)	n.s.
Long gap (≥3 vertebrae)	92%	100%	n.s.
Vogt Type 3b	46%	46%	n.s.
Tracheoesophageal fistula	46%	62%	n.s.

Comparison was conducted using chi-square tests or *t*-tests. Significance level is set at *p* < 0.05

*n.s*. not significant

**Table 2:** Distribution of clinical outcomes of patients who underwent repair via secondary anastomosis: delayed primary anastomosis or gastric sleeve pull-up

	Delayed primary anastomosis *(n *= 13*)*	Gastric sleeve pull-up (*n *= 13)	*p*
Clinical outcomes	Mean (SD)	Mean (SD)	
Anastomosis surgery			
Prior surgeries (*n*)	1.00 (0.00)	1.38 (0.65)	0.04
Age on day of surgery (days)	128.64 (114.63)	141.15 (106.04)	n.s.
Weight on day of surgery (g)	4153.83 (1694.55)	5032.50 (1373.88)	n.s.
Duration of surgery (min)	266.73 (217.83)	341.33 (88.19)	n.s.
Duration of general anesthesia (min)	328.82 (88.54)	478.54 (98.93)	< 0.001
Post-operative details			
Length of stay in ICU (days)	25.18 (19.49)	15.77 (17.43)	n.s.
Duration of mechanical ventilation (days)	11.17 (9.96)	5.77 (4.00)	n.s.
Presence of chest tube	91%	92%	n.s.
Duration of chest tube (days)	9.00 (6.23)	8.64 (4.22)	n.s.
Presence of trans-anastomotic feeding tube	91%	100%	n.s.
Complications			
Leakage	15%	23%	n.s.
Re-fistula	0%	0%	n.s.
Tracheomalacia	62%	69%	n.s.
Duration of tracheomalacia symptoms (months)	17.31 (25.38)	12.00 (18.18)	n.s.
Scoliosis	15%	0%	n.s.
Recurrent respiratory infections	67%	69%	n.s.
Admissions due to respiratory infections (*n*)	3.50 (3.29)	3.46 (3.31)	n.s.
Dysphagia	52%	62%	n.s.
Gastroesophageal reflux	54%	46%	n.s.
Duration of gastroesophageal reflux (months)	23.38 (30.25)	7.42 (12.04)	n.s.
GERD-Q score	8.17 (4.92)	4.83 (4.31)	n.s.
Use of PPI medication (months)	81.67 (146.61)	19.85 (19.99)	n.s.
Stricture	62%	69%	n.s.
Dilatation procedures			
Endoscopic dilatations required	69%	100%	0.03
Endoscopic dilatations (*n*)	9.46 (11.04)	13.69 (28.92)	n.s.
Duration of anesthesia for dilatation procedures (min)	630.15 (861.89)	523.54 (1074.43)	n.s.
Duration of hospitalization for dilatation procedures (days)	15.23 (31.84)	14.38 (18.21)	n.s.
Feeding			
Time until full oral feeding post-anastomosis (days)	532.38 (683.75)	240.38 (138.91)	n.s.
FOIS: full feeding w/o restrictions at time of data acquisition	57%	17%	n.s.
Gastric tube	100%	0%	< 0.001
Jejunal tube	38%	100%	< 0.001
Duration of feeding through feeding tube (days)	669.62 (760.47)	389.77 (309.29)	n.s.
Duration TPN (days)	31.92 (66.68)	13.46 (26.53)	n.s.
Esophageal atresia-related surgeries and hospitalizations			
Cumulative duration of general anesthesia (min)	1567.00 (1207.37)	1444.92 (1260.97)	n.s.
Cumulative operating time (minutes)	718.46 (534.46)	708.69 (487.96)	n.s.
Cumulative hospitalizations (days)	214.00 (118.02)	172.69 (105.91)	n.s.
Cumulative length of stay in the ICU (days)	94.75 (69.36)	42.31 (43.90)	0.03
Mortality	31%	0%	0.03

Comparison between the groups was conducted using Chi-square tests or *t*-tests. Significance level is set at *p* < 0.05

*n.s*. not significant

### Clinical outcome of delayed anastomosis vs. gastric pull-up

As presented in Table 3, there were no significant differences regarding gap length between the secondary anastomosis subgroups. Additionally, as patients were propensity score matched for gender, age, and weight at the time of surgery, there were no significant differences with regards to these factors (Table 4). As summarized in Table 4, there were no differences regarding clinical outcomes and long-term complications between the two secondary anastomosis subgroups. However, the duration of general anesthesia during the surgery was significantly higher in the gastric sleeve pull-up group than the delayed anastomosis group. When analyzing all atresia-related surgeries and hospitalizations, no significant differences in the cumulative duration of general anesthesia or operating time between these two groups were found. Additionally, there were no significant differences regarding the of number of days spent in ICU or the duration of machine ventilation time post-operatively. Even more, the rates of post-operative complications, such as anastomosis leakage, strictures, re-fistula, tracheomalacia, scoliosis, recurrent infections or reflux (i.e., functional oral intake scale) were similar across both groups. The cumulative number of days spent in the ICU immediately following anastomosis surgery and all stays related to the atresia diagnosis thereafter was referred to as ‘cumulative length of stay in the ICU’, which was significantly higher in the delayed primary anastomosis group. The overall mortality rate was significantly lower in the gastric sleeve pull-up group. However, bearing in mind that patients in the gastric sleeve pull-up group were less frequent pre-term and had a lower rate of cardiac and anorectal malformations could potentially be accounting for this significant difference in mortality. As shown in Fig. 1, there were no long-term significant differences in weight gain between the gastric sleeve pull-up and delayed primary anastomosis group.

**Table 3:** Distribution of disease characteristics of the patient cohort, grouped according to the method of EA repair: primary versus secondary anastomosis

	Primary anastomosis *(n=61)*	Secondary anastomosis *(n=37)*	*p*
Disease characteristics	Mean (SD)	Mean (SD)	
Demographics			
Gender (female)	39%	41%	n.s.
Gestation week	36.84 (3.33)	33.59 (3.81)	<.001
Birth weight (g)	2575.95 (737.10)	1918.08 (856.82)	<.001
Birth weight (z-score)	− 1.00 (0.99)	− 1.02 (1.04)	n.s.
Body length (cm)	47.71 (5.07)	43.48 (5.22)	<.001
Body length (z-score)	− 0.76 (0.91)	− 0.64 (1.11)	n.s.
Gemini	3%	22%	.004
Preterm (<36 gestational week)	30%	70%	<.001
Cesarean-section	59%	83%	.01
Family history of esophageal atresia	2%	0%	n.s.
Presence of associated malformations			
Cardiac malformation	56%	49%	n.s.
Anorectal malformation	7%	19%	n.s.
Urogenital malformation	18%	24%	n.s.
Gastrointestinal malformation	7%	24%	.01
Musculoskeletal malformation	23%	24%	n.s.
Esophageal atresia			
Distance measured between proximal and distal ends (vertebrae)	2.12 (0.84)	3.55 (1.67)	<.001
Long gap (≥3 vertebrae)	18%	63%	<.001
Vogt type 3b	92%	76%	<.001
Tracheoesophageal fistula	98%	57%	<.001

Comparison of the groups was conducted using chi-square tests or *t*-tests. Significance level is set at *p* < 0.05

*n.s*. not significant

**Table 4:** Distribution of clinical outcomes of the patient cohort according to the method of EA repair; primary or secondary anastomosis

	Primary anastomosis *(n *= 61*)*	Secondary anastomosis *(n *= 37*)*	*p*
Clinical outcomes	Mean (SD)	Mean (SD)	
Anastomosis surgery			
Prior operations (*n*)	0.00 (0.00)	1.14 (0.42)	< 0.001
Age on day of surgery (days)	4.85 (22.90)	122.48 (94.77)	< 0.001
Weight on day of surgery (g)	2566.98 (801.86)	4183.58 (1598.13)	< 0.001
Duration of surgery (min)	120.53 (28.35)	282.86 (154.27)	< 0.001
Time under anesthetic (min)	229.71 (62.69)	390.57 (116.22)	< 0.001
Post-surgical details			
Length of stay in ICU (days)	15.37 (24.41)	20.19 (19.09)	n.s.
Duration of mechanical ventilation (days)	7.80 (15.06)	8.18 (7.73)	n.s.
Presence of chest tube	44%	93%	< 0.001
Duration of chest tube (days)	3.14 (5.06)	8.71 (4.81)	< 0.001
Presence of trans-anastomotic feeding tube	98%	97%	n.s.
Complications			
Leakage	7%	19%	0.04
Re-fistula	0%	3%	n.s.
Tracheomalacia	38%	60%	0.03
Duration of tracheomalacia symptoms (months)	12.63 (25.48)	12.43 (19.99)	n.s.
Scoliosis	14%	9%	n.s.
Recurrent respiratory infections	27%	82%	n.s.
Admissions due to respiratory infections (*n*)	2.26 (2.67)	3.21 (3.51)	n.s.
Dysphagia	39%	63%	0.04
Gastroesophageal reflux	43%	60%	n.s.
Duration of gastroesophageal reflux (months)	13.10 (29.52)	16.73 (22.68)	n.s.
GERD-Q score	2.94 (3.15)	6.50 (4.74)	0.02
Use of PPI medications (months)	17.63 (30.50)	46.71 (91.91)	0.039
Fundoplication	2%	12%	0.017
Stricture	39%	62%	0.028
Dilatation procedures			
Endoscopic dilatations required	43%	79%	< 0.001
Endoscopic dilatations (*n*)	2.13 (5.18)	9.76 (19.11)	0.004
Duration of anesthesia for dilation procedures (min)	123.22 (328.10)	491.62 (851.56)	0.004
Duration of hospitalization for dilation procedures (days)	3.29 (5.36)	7.88 (7.46)	n.s.
Feeding			
Time until full oral feeding post-anastomosis (days)	91.83 (291.13)	362.05 (431.99)	0.003
FOIS: full feeding w/o restrictions at time of data acquisition	76%	38%	0.04
Gastric tube	18%	62%	< 0.001
Jejunal tube	5%	59%	< 0.001
Duration of feeding through tube feeding (days)	29.23 (50.39)	13.97 (16.04)	n.s.
Duration TPN (days)	4.49 (7.18)	18.00 (43.87)	0.02
Esophageal atresia-related surgeries and hospitalizations			
Cumulative duration of general anesthesia (min)	483.31 (476.95)	1318.26 (1120.98)	< 0.001
Cumulative operating time (min)	224.88 (236.49)	629.34 (468.35)	< 0.001
Cumulative hospitalizations (days)	59.21 (56.72)	175.69 (105.33)	< 0.001
Cumulative length of stay in the ICU (days)	20.38 (28.03)	64.53 (55.48)	< 0.001
Mortality	8%	16%	n.s.

Comparison of the groups is conducted with Chi-square test or* t*-tests. Significance level is set at *p* < 0.05

*n.s*. not significant

### (Health-related) Quality of life and mental health

Out of 104 families assessed for eligibility and 63 families who received the questionnaires, in total 39 families responded and were included in analysis of the questionnaires. Thus, responder rate was almost comparable to previous studies [16, 17]. There were no significant differences between respondents and non-respondents regarding weight, age, gender, gap length, associated co-morbidities, operation method and complications. Supplementary Fig. 2 shows the CONSORT flow diagram illustrating the total patient cohort for analysis of HRQoL and mental health questionnaires. Table 5 shows the sociodemographic and disease characteristics of the participating parents and their affected child. When comparing primary with secondary anastomosis, as well as delayed primary anastomosis with gastric sleeve pull-up, there were no significant differences. Neither the QoL and mental health of the patient’s parents, nor the parent-reported HRQoL and mental health of the affected child, showed any significant differences in the comparison of these groups. Additionally, the entire cohort was analyzed in comparison to norm data available for each of the standardized questionnaires. Here, mothers reported a significant reduction in their child’s HRQoL and mental health, while fathers only reported a significant reduction in their child’s mental health. Data is summarized in Table 6.

**Table 5.** Distribution of the reported sociodemographic and disease characteristics of the patient cohort

	Patient cohort (*n*=39)	
Characteristics	Mean	SD
Patient’s age (years)	5.3	4.40
Mother’s age (years)	38.5	7.01
Father’s age (years)	41.1	5.12
Number of children in family	1.7	0.61
Number of surgeries due to disease	6.5	6.69
Time since last surgery (years)	3.0	3.33
Time since first surgery (years)	5.3	4.40

Parents	*n*	%
Parent’s gender (mothers/fathers)	37/25	94.9/64.1
Marital status (mothers/fathers)		
Married/living together	28/22	71.8/56.4
Single	5/2	12.8/5.1
Divorced	2/1	5.1/2.6
Not stated	2/0	5.1/0.0
Education (mothers/fathers)		
Lower-middle education	14/9	35.9/23.1
Higher education	18/14	46.2/35.9
Not stated	3/2	7.7/5.1
Employment^a^ (mothers/fathers)		
Fully employed	7/19	17.9/48.7
Partly employed	15/3	38.5/7.7
No employment	2/2	5.2/5.2
Not stated	1/1	2.6/2.6

Patients	*n*	%
Patient's gender		
Female	16	41.0
Male	23	59.0
Patient receives level of care^b^		
Yes	20	51.3
No	15	38.5
VACTERL Association		
Yes	12	30.8
No	27	69.2

^a^Refers to the last 12 months

^b^Refers to the decision for the classification in the care insurance according to the German long-term care insurance

**Table 6:** Distribution of parental QoL and mental health, and parent-reported HrQoL and mental health for children after primary anastomosis (b), secondary anastomosis (c) as well as delayed primary anastomosis (d) and gastric sleeve pull-up (e).

	Total cohort (a)	Primary anastomosis (b)	Secondary anastomosis (c)	Delayed primary anastomosis (d)	Gastric sleeve pull-up (e)	Norm data (f)	(*p*) effect size a vs. f	(*p*) effect size b vs. c	(*p*) effect size d vs. e	Total cohort (a)	Primary anastomosis (b)	Secondary anastomosis (c)	Delayed primary anastomosis (d)	Gastric sleeve pull-up (e)	Norm data (f)	(*p*) effect size a vs. f	(*p*) effect size b vs. c	(*p*) effect size d vs. e	Total cohort (a)	Primary anastomosis (b)
	Mdn	M	SD	Mdn	M	SD	Mdn	M	SD	Mdn	M	SD	Mdn	M	SD	M	SD			
Mothers																				
QoL	2.6	2.6	0.53	2.6	2.6	0.61	2.7	2.6	0.44	2.7	2.6	0.55	2.5	2.6	0.51	2.6	0.53	(.68) 0.07	(.69) 0.13	(.86) 0.12
Mental health	0.3	0.4	0.38	0.3	0.5	0.46	0.3	0.4	0.36	0.3	0.5	0.41	0.3	0.3	0.27	0.4	0.23	(.29) 0.18	(.77) 0.10	(.69) 0.25
Children’s HrQoL	79.8	79.9	13.17	81.7	80.8	12.50	79.8	77.8	14.81	81.3	79.0	12.85	78.3	75.7	17.31	86.1	11.2	(.01) 0.47	(.63) 0.17	(.90) 0.10
Children’s mental health	11.0	11.6	3.72	10.0	11.0	3.29	12.0	12.7	4.21	13.0	13.6	4.82	11.0	11.4	3.21	8.5	7.22	(<.01) 0.82	(.15) 0.71	(.43) 0.94
Fathers																				
QoL	2.8	2.8	0.55	2.8	2.8	0.44	2.7	2.7	0.75	2.7	2.8	0.77	2.2	2.2	0.9	2.6	0.53	(.21) 0.27	(.34) 0.38	(.50) 0.84
Mental health	0.3	0.3	0.47	0.2	0.2	0.22	0.3	0.5	0.71	0.3	0.3	0.24	1.2	1.18	1.61	0.3	0.23	(.52) 0.13	(.29) 0.42	(.89) 0.20
Children’s HrQoL	88.3	84.0	12.80	88.3	83.3	13.81	88.3	85.7	10.81	79.9	81.1	9.57	95.0	95.0	7.07	86.1	11.2	(.46) 0.16	(.70) 0.17	(.27) 1.38
Children’s mental health	10.0	10.4	2.99	10.0	10.5	3.16	11.5	10.0	2.76	12.0	10.8	2.5	8.5	8.5	3.54	8.5	7.22	(<.01) 0.62	(.99) 0.01	(.27) 1.38

*QoL *quality of life, *HrQoL* Health-related quality of life, *Mdn* median, *M m*ean, *SD*= standard deviation

The psychosocial constructs are represented by the following questionnaires: QoL = ULQIE, Mental health = BSI, Children’s HRQoL = PedsQL TM 4.0 SF-15, Children’s mental health = SDQ. Comparison between groups was conducted using Welch *t*-test and one-sample *t*-test. *d* = Cohen’s *d*. Significance level was set at *p* < 0.05

## Discussion

The debate amongst pediatric surgeons whether to preserve or replace the esophagus in LGEA patients has been ongoing for decades [9, 10]. Proponents of a delayed anastomosis procedure often state that spontaneous growth between the atretic ends of the esophagus can occur in children with EA within the first months of their lives. Therefore, waiting a specific time before performing corrective EA surgery is favored. Indeed, three months after birth the newborn’s esophagus is much thicker and more resilient than directly after birth [33]. To accelerate this process, some surgeons advocate elongation of the esophagus under traction (Foker technique) [34]. This technique has, however, been associated with a high rate of esophageal strictures and stump tears [35]. On the other hand, proponents of the primary gastric sleeve pull-up argue that preservation is only useful if the preserved esophagus is functioning properly, which may only be true for “shorter” long gaps [36]. However, the primary gastric sleeve pull-up procedure is irreversible and disrupts gastrointestinal physiology. As such, malignancy rates associated with chronic reflux need to be considered as a long-term complication. Chronic GERD after EA can lead to mucosal damage, esophageal structuring, Barrett’s esophagus, and eventually esophageal adenocarcinoma. Higher incidences of these complications have been reported in adults after EA repair, regardless of the technique [37, 38].

The current study suggests that the outcome after gastric sleeve pull-up (replacement) and delayed primary anastomosis (preservation) in LGEA patients is comparable in many aspects. This was observed regarding anastomosis leakage, stricture rate, reflux, dysphagia, and cumulative operating time. Yet significant differences were found in the cumulative duration of stays in the ICU as well as mortality which may be heavy affected by associated malformations. However, HrQoL and mental health of the patients as well as their parents were found to be similar in both subgroups.

With respect to clinical outcomes, our patient cohort showed a high rate of dysphagia and reflux in both secondary anastomosis subgroups. However, this outcome is not uncommon and has been reported as a common long-term complication following repair surgery in LGEA patients [39]. Yet, when examining dysphagia and reflux amongst patients who underwent the primary as well as secondary anastomosis approach in our study, our findings were able to confirm those of a recent study. This study revealed that patients who underwent early definitive repair of the esophagus reported significantly lower incidences of oro-pharyngeal dysphagia [40].

With respect to the HRQoL, digestive issues, such as the ones mentioned above, have been shown to significantly impact patients and their family’s HRQoL [41–43]. On the bright side however, studies have reported that adults who underwent EA surgery as children do not report a lower HRQoL because of this reflux or dysphagia [44, 45]. The current study, however, showed a significant reduction in parent-reported HRQoL and mental health of patients with EA when compared to norm values, which is in line with a recent report [46]. When examining the different EA treatment approaches, no significant differences in the parent-reported HRQoL or mental health of the children were found, while parents themselves, also reported no significant differences in their own HRQoL or mental health. One explanation for these contradictory findings can possibly be explained by the fact that in our study parents filled out the forms on behalf of their respective child and the patients themselves were not asked to participate. This might skew the actual HRQoL of the individuals treated for EA, as it has been described that proxies may report the HRQoL poorer than EA patients themselves [47].

Summing up, when considering which surgical procedure to perform in order to treat LGEA, every surgeon should not only weigh the risks of the surgery, but also the risks not directly associated with the actual surgical procedure. As an example, it has been proposed that the newborn stage coincides with a time frame of rapid brain development. During this time, surgery performed with concomitant anesthesia may disrupt very important stages of development. It has been reported that complex surgeries and long anesthesia may lead to neurodevelopmental delays in cognition, learning, and behavior [48]. Recently, long-term neurodevelopment impairment in children with EA have been found; especially in motor function and in cognitive performance [49].

Consequently, multiple interventions, with or without general anesthesia, ought to be avoided in the neonatal period and should be considered when developing a treatment plan for a patient with LGEA. A single rather than multiple operations positively affect HRQoL [50]. As such, factors such as HRQoL and mental health should be considered key essentials in determining which surgical technique is ‘superior’.

### Limitations

Most limitations of the current study are inherent with the retrospective cross-sectional study design, in particular the small numbers and the lack of randomization. Thus, propensity score matching was used to limit the effects of the most relevant factors [51]. Moreover, due to the cross-sectional nature of the design, the statements regarding the treatment evaluation can only be made cautiously. The rate of non-respondents for the HRQoL and mental health assessment is unfortunately frequent and therefore may affect the results [52]. Even though a condition-specific questionnaire is recommended in addition to the use of a general questionnaire for HRQoL in previous research as a condition-specific questionnaire is generally more sensitive to evaluate clinical differences [53–56], we did not use it due to the total amount of questionnaires used in the research project, which should be considered as a limitation of our study. Nonetheless, validated standardized instruments were used, which is a strength of the study. Moreover, GERD-Q is often used but has not been validated for children. Thus, reflux may be either over- or underestimated in the current study. Finally, in this study, parent-proxy instead of child-report has been used which may over- or under-estimate certain aspects.

## Conclusion

Based on the results of the current study, patients with gastric sleeve pull-up procedure have similar clinical outcomes, generic HRQoL and mental health when compared to delayed primary anastomosis. However, the long-term results of either preservation or replacement of the esophagus remain uncertain, particularly regarding chronic reflux-associated metaplasia resulting in esophageal adenocarcinoma, neurodevelopmental impact of multiple surgeries, condition-specific QoL and needs of follow-up care. Future studies should focus on these aspects.

### Supplementary Information

Below is the link to the electronic supplementary material.

Supplementary file 1 CONSORT Flow Diagram illustrating the total patient cohort and subgroups for analysis of clinical outcomes.

Supplementary file 2 CONSORT Flow Diagram illustrating the total patient cohort for analysis of HRQoL and mental health questionnaires.

## Data Availability

All data is avaiable upon request of the corresponding author.

## References

[1] Nassar N, Leoncini E, Amar E (2012). Prevalence of esophageal atresia among 18 international birth defects surveillance programs. Birth Defects Res A Clin Mol Teratol.

[2] Elrod J, Boettcher M, Mohr C, Reinshagen K (2021). An analysis of the care structure for congenital malformations in Germany. Dtsch Arztebl Int.

[3] Stadil T, Koivusalo A, Svensson JF (2019). Surgical treatment and major complications within the first year of life in newborns with long-gap esophageal atresia gross type A and B—a systematic review. J Pediatr Surg.

[4] Baird R, Lal DR, Ricca RL (2019). Management of long gap esophageal atresia: a systematic review and evidence-based guidelines from the APSA Outcomes and Evidence Based Practice Committee. J Pediatr Surg.

[5] Tan Tanny SP, Comella A, Hutson JM (2019). Quality of life assessment in esophageal atresia patients: a systematic review focusing on long-gap esophageal atresia. J Pediatr Surg.

[6] Shieh HF, Jennings RW (2017). Long-gap esophageal atresia. Semin Pediatr Surg.

[7] Subramaniam T, Martin BP, Jester I (2022). A single centre experience using internal traction sutures in managing long gap oesophageal atresia. J Pediatr Surg..

[8] Patkowski D, Lacher M, Muensterer OJ (2021). Thoracoscopic technique using internal traction sutures for long-gap esophageal atresia repair. Video atlas of pediatric endosurgery (VAPE).

[9] Lee HQ, Hawley A, Doak J (2014). Long-gap oesophageal atresia: comparison of delayed primary anastomosis and oesophageal replacement with gastric tube. J Pediatr Surg.

[10] Jensen AR, McDuffie LA, Groh EM, Rescorla FJ (2020). Outcomes for correction of long-gap esophageal atresia: a 22-year experience. J Surg Res.

[11] Hannon E, Eaton S, Curry JI (2020). Outcomes in adulthood of gastric transposition for complex and long gap esophageal atresia. J Pediatr Surg.

[12] Ebrahim S (1995). Clinical and public health perspectives and applications of health-related quality of life measurement. Soc Sci Med.

[13] Galderisi S, Heinz A, Kastrup M (2015). Toward a new definition of mental health. World Psychiatry.

[14] The World Health Organization Quality of Life Group (1995). The World Health Organization quality of life assessment (WHOQOL): Position paper from the World Health Organization. Soc Sci Med.

[15] Witt S, Dingemann J, Dellenmark-Blom M, Quitmann J (2021). Parent–child assessment of strengths and difficulties of german children and adolescents born with esophageal atresia. Front Pediatr.

[16] Mikkelsen A, Boye B, Diseth TH (2022). Traumatic stress, mental health, and quality of life in adolescents with esophageal atresia. J Pediatr Surg.

[17] Dellenmark-Blom M, Ax SÖ, Lilja HE (2022). Prevalence of mental health problems, associated factors, and health-related quality of life in children with long-gap esophageal atresia in Sweden. J Pediatr Surg.

[18] Dellenmark-Blom M, ÖrnöAx S, Öst E (2022). Postoperative morbidity and health-related quality of life in children with delayed reconstruction of esophageal atresia: a nationwide Swedish study. Orphanet J Rare Dis.

[19] Gallo G, van Tuyll van Serooskerken ES, Tytgat SHAJ (2021). Quality of life after esophageal replacement in children. J Pediatr Surg.

[20] Youn JK, Park T, Kim SH (2018). Prospective evaluation of clinical outcomes and quality of life after gastric tube interposition as esophageal reconstruction in children. Medicine.

[21] Witt S, Dellenmark-Blom M, Dingemann J (2019). Quality of life in parents of children born with esophageal atresia. Eur J Pediatr Surg.

[22] Tan Tanny SP, Trajanovska M, Muscara F (2021). Quality of life outcomes in primary caregivers of children with esophageal atresia. J Pediatr.

[23] Vandenbroucke JP, von Elm E, Altman DG (2007). Strengthening the reporting of observational studies in epidemiology (STROBE): explanation and elaboration. Epidemiology.

[24] Crary MA, Carnaby Mann GD, Groher ME (2005). Initial psychometric assessment of a functional oral intake scale for dysphagia in stroke patients. Arch Phys Med Rehabil.

[25] IJsselstijn H, Gischler SJ, Toussaint L (2016). Growth and development after oesophageal atresia surgery: need for long-term multidisciplinary follow-up. Paediatr Respir Rev.

[26] Jonasson C, Wernersson B, Hoff DAL, Hatlebakk JG (2013). Validation of the GerdQ questionnaire for the diagnosis of gastro-oesophageal reflux disease. Aliment Pharmacol Ther.

[27] Jones R, Junghard O, Dent J (2009). Development of the GerdQ, a tool for the diagnosis and management of gastro-oesophageal reflux disease in primary care. Aliment Pharmacol Ther.

[28] Goldbeck L, Storck M (2002). Das Ulmer Lebensqualitäts-Inventar für Eltern chronisch kranker Kinder (ULQIE). Z Klin Psychol Psychother.

[29] Geisheim C, Hahlweg K, Fiegenbaum W (2002). Das brief symptom inventory (BSI) als Instrument zur Qualitätssicherung in der Psychotherapie. Diagnostica.

[30] Chan KS, Mangione-Smith R, Burwinkle TM (2005). The PedsQL: reliability and validity of the short-form generic core scales and asthma module. Med Care.

[31] Felder-Puig R, Frey E, Proksch K (2004). Validation of the German version of the Pediatric Quality of Life Inventory (PedsQL) in childhood cancer patients off treatment and children with epilepsy. Qual Life Res.

[32] Klasen H, Woerner W, Rothenberger A, Goodman R (2003) Die deutsche Fassung des Strengths and Difficulties Questionnaire (SDQ-Deu)-Übersicht und Bewertung erster Validierungs-und Normierungsbefunde. Prax Kinderpsychol Kinderpsychiatr 52:491–502. http://hdl.handle.net/20.500.11780/2700 14526759

[33] Oliver DH, Martin S, Belkis DMI (2021). Favorable outcome of electively delayed elongation procedure in long-gap esophageal atresia. Front Surg.

[34] Krishnan U, Singh H, Kaakoush N (2019). DOZ047.19: outcomes in the management of long-gap esophageal atresia: is the Foker technique superior?. Diseases Esophagus.

[35] Nasr A, Langer J (2013). Mechanical traction techniques for long-gap esophageal atresia: a critical appraisal. Eur J Pediatr Surg.

[36] Zeng Z, Liu F, Ma J (2017). Outcomes of primary gastric transposition for long-gap esophageal atresia in neonates. Medicine.

[37] Taylor ACF, Breen KJ, Auldist A (2007). Gastroesophageal reflux and related pathology in adults who were born with esophageal atresia: a long-term follow-up study. Clin Gastroenterol Hepatol.

[38] Sistonen SJ, Koivusalo A, Nieminen U (2010). Esophageal morbidity and function in adults with repaired esophageal atresia with tracheoesophageal fistula: a population-based long-term follow-up. Ann Surg.

[39] Hölscher AC, Laschat M, Choinitzki V (2017). Quality of life after surgical treatment for esophageal atresia: long-term outcome of 154 patients. Eur J Pediatr Surg.

[40] Soyer T, Arslan SS, Boybeyi Ö (2022). The role of oral feeding time and sham feeding on oropharyngeal swallowing functions in children with esophageal atresia. Dysphagia.

[41] Dellenmark-Blom M, Quitmann J, Dingemann J (2020). Clinical Factors Affecting Condition-Specific Quality-of-Life Domains in Pediatric Patients after Repair of Esophageal Atresia: The Swedish-German EA-QOL Study. Eur J Pediatr Surg.

[42] di Natale A, Brestel J, Mauracher AA (2022). Long-term outcomes and health-related quality of life in a Swiss patient group with esophageal atresia. Eur J Pediatr Surg.

[43] Rozensztrauch A, Śmigiel R, Błoch M, Patkowski D (2020). The impact of congenital esophageal atresia on the family functioning. J Pediatr Nurs.

[44] Tannuri U, Tannuri ACA, Gonçalves MEP, Cardoso SR (2008). Total gastric transposition is better than partial gastric tube esophagoplasty for esophageal replacement in children. Dis Esophagus.

[45] Deurloo JA, Ekkelkamp S, Hartman EE (2005). Quality of life in adult survivors of correction of esophageal atresia. Arch Surg.

[46] Dellenmark-Blom M, Ax SÖ, Lilja HE (2023). Prevalence of mental health problems, associated factors, and health-related quality of life in children with long-gap esophageal atresia in Sweden. J Pediatr Surg.

[47] Witt S, Bloemeke J, Bullinger M (2019). Agreement between mothers’, fathers’, and children’s’ ratings on health-related quality of life in children born with esophageal atresia—a German cross-sectional study. BMC Pediatr.

[48] Keunen K, Sperna Weiland NH, de Bakker BS (2022). Impact of surgery and anesthesia during early brain development: a perfect storm. Paediatr Anaesth.

[49] van Hoorn CE, ten Kate CA, Rietman AB (2021). Long-term neurodevelopment in children born with esophageal atresia: a systematic review. Dis Esophagus.

[50] Ludman L, Spitz L (2003). Quality of life after gastric transposition for oesophageal atresia. J Pediatr Surg.

[51] Haukoos JS, Lewis RJ (2015). The propensity score. JAMA.

[52] Coste J, Quinquis L, Audureau E, Pouchot J (2013). Non response, incomplete and inconsistent responses to self-administered health-related quality of life measures in the general population: patterns, determinants and impact on the validity of estimates—a population-based study in France using the MOS SF-36. Health Qual Life Outcomes.

[53] Dellenmark-Blom M, Chaplin JE, Gatzinsky V (2016). Health-related quality of life experiences among children and adolescents born with esophageal atresia: development of a condition-specific questionnaire for pediatric patients. J Pediatr Surg.

[54] Dellenmark-Blom M, Dingemann J, Witt S (2018). The esophageal-atresia-quality-of-life questionnaires: feasibility, validity and reliability in Sweden and Germany. J Pediatr Gastroenterol Nutr.

[55] ten Kate CA, Rietman AB, Kamphuis LS (2021). Patient-driven healthcare recommendations for adults with esophageal atresia and their families. J Pediatr Surg.

[56] TenKate CA, Teunissen NM, Group on behalf of the DS (2022). Development and validation of a condition-specific quality of life instrument for adults with esophageal atresia: the SQEA questionnaire. Diseases Esophagus.

