# Supplementary material for: Clinical outcome, quality of life, and mental health in long-gap esophageal atresia: comparison of gastric sleeve pull-up and delayed primary anastomosis
Source: Pediatr Surg Int. 2023 Apr 4;39(1):166. doi: 10.1007/s00383-023-05448-4 (PMC10073059; doi:10.1007/s00383-023-05448-4)
Supplement: Supplementary file 1 — CONSORT Flow Diagram illustrating the total patient cohort and subgroups for analysis of clinical outcomes. [file 383_2023_5448_MOESM1_ESM.docx]

**Supplementary Figure 1**: CONSORT Flow Diagram illustrating the total patient cohort and subgroups for analysis of clinical outcomes

**Gastric sleeve pull-up (*n*=13)**

**Delayed Primary Anastomosis (*n*=13)**

Gastric sleeve pull-up (*n*=13)

Delayed Primary Anastomosis (*n*=23)

**Excluded (*n*=1)**
Not meeting inclusion criteria (n=1)

**Primary Anastomosis (*n*=61)**

**Secondary Anastomosis (*n*= 37)**

Propensity Score Matching
*(gender, age, weight at operation, diagnosis and revisionary surgery)*

**Included (*n*=98)**

**Assessed for eligibility (*n*=104)**

**Excluded (*n*= 6)**

Not meeting inclusion criteria (*n*=5)

Declined to participate (*n*=1)
