# Supplementary material for: Clinical outcome, quality of life, and mental health in long-gap esophageal atresia: comparison of gastric sleeve pull-up and delayed primary anastomosis
Source: Pediatr Surg Int. 2023 Apr 4;39(1):166. doi: 10.1007/s00383-023-05448-4 (PMC10073059; doi:10.1007/s00383-023-05448-4)
Supplement: Supplementary file 2 — CONSORT Flow Diagram illustrating the total patient cohort for analysis of HRQoL and mental health questionnaires. [file 383_2023_5448_MOESM2_ESM.docx]

**Supplementary Figure 2**: CONSORT Flow Diagram illustrating the total patient cohort for analysis of QoL and mental health questionnaires

**Assessed for eligibility (*n*= 104)**

**Received questionnaires (*n*= 63)**

**Analyzed (*n*= 39)**

**Excluded** (*n*= 41)

Not meeting inclusion criteria (*n*= 5)
Declined to participate (*n*= 1)

Contact data out of date (*n*= 32)

Lack of German skills (*n*=3)

**Excluded** (*n*=24)

Non respondent (*n*=24)
